# 247. The Predictive Value of Methicillin-Resistant *Staphylococcus aureus* Surveillance Swabs in Septic Arthritis

**DOI:** 10.1093/ofid/ofab466.449

**Published:** 2021-12-04

**Authors:** Samuel Harder, Kwame Asiamah, Geoffrey Shumilak, Beverly J Wudel

**Affiliations:** 1 University of Saskatchewan College of Medicine, Saskatoon, Saskatchewan, Canada; 2 University of Saskatchewan, Saskatoon, Saskatchewan, Canada

## Abstract

**Background:**

Septic arthritis is a destructive form of acute arthritis secondary to infection. With an annual incidence of 2 to 5 cases per 100 000 individuals, it is associated with significant morbidity and mortality. Prompt source control and antimicrobial therapy remain the mainstays of management. Epidemiology, microbiology studies, and local resistance patterns are important in guiding therapeutic decisions. Staphylococcal and streptococcal species are the most common pathogens with Methicillin-resistant Staphylococcus aureus (MRSA) becoming an increasingly important pathogen. The increasing incidence of MRSA provides clinicians with the challenge of deciding which patients require empiric coverage for MRSA. MRSA nasal screening has been shown to have a high negative predictive value in pneumonia, bloodstream infections, and nosocomial infections in critically ill patients. However, little is known about the diagnostic utility of MRSA surveillance swabs for predicting MRSA infections in septic arthritis.

**Methods:**

A retrospective cohort study was performed in 3 tertiary hospitals from September 1, 2010 to December 31, 2020. All adult patients with confirmed septic arthritis of the ankle, wrist, knee, or hip and an MRSA surveillance swab performed within 72 hours of admission were included in the study. These data were used to calculate the sensitivity, specificity, positive predictive value and negative predictive value for MRSA surveillance swabs.

**Results:**

One hundred seventy-two patients met inclusion criteria. Thirty patients had positive MRSA surveillance swabs. The prevalence of MRSA in joint cultures was 11.04%. The positive predictive value of MRSA surveillance swabs was 42.3% and the negative predictive value was 93.5% in all participants. The MRSA surveillance swab had a negative predictive value of 100% in participants with no risk factors for MRSA colonization.

**Conclusion:**

The negative predictive value of MRSA surveillance swabs used independently is insufficient to confidently rule out MRSA as the causative pathogen in septic arthritis. When used in combination with MRSA risk factors, the absence of MRSA risk factors may help clinicians rule out MRSA as a causative pathogen.

**Disclosures:**

**All Authors**: No reported disclosures

